# The effects of the novel A53E alpha-synuclein mutation on its oligomerization and aggregation

**DOI:** 10.1186/s40478-016-0402-8

**Published:** 2016-12-09

**Authors:** Diana F. Lázaro, Mariana Castro Dias, Anita Carija, Susanna Navarro, Carolina Silva Madaleno, Sandra Tenreiro, Salvador Ventura, Tiago F. Outeiro

**Affiliations:** 1 Department of Neurodegeneration and Restorative Research, University Medical Center Göttingen, Waldweg 33, 37073 Göttingen, Germany; 2 Institut de Biotecnologia i Biomedicina and Departament de Bioquímica i Biologia Molecular, Universitat Autònoma de Barcelona, 08193 Bellaterra Barcelona, Spain; 3 Chronic Disease Research Center (CEDOC), NOVA Medical School, Campo dos Mártires da Pátria, 130, 1169-056 Lisbon, Portugal; 4 Max Planck Institute for Experimental Medicine, Göttingen, Germany

**Keywords:** Alpha-synuclein, Parkinson’s disease, Oligomerization, Aggregation, Neurodegeneration

## Abstract

α-synuclein (aSyn) is associated with both sporadic and familial forms of Parkinson’s disease (PD), the second most common neurodegenerative disorder after Alzheimer’s disease. In particular, multiplications and point mutations in the gene encoding for aSyn cause familial forms of PD. Moreover, the accumulation of aSyn in Lewy Bodies and Lewy neurites in disorders such as PD, dementia with Lewy bodies, or multiple system atrophy, suggests aSyn misfolding and aggregation plays an important role in these disorders, collectively known as synucleinopathies. The exact function of aSyn remains unclear, but it is known to be associated with vesicles and membranes, and to have an impact on important cellular functions such as intracellular trafficking and protein degradation systems, leading to cellular pathologies that can be readily studied in cell-based models. Thus, understanding the molecular effects of aSyn point mutations may provide important insight into the molecular mechanisms underlying disease onset.

We investigated the effect of the recently identified A53E aSyn mutation. Combining in vitro studies with studies in cell models, we found that this mutation reduces aSyn aggregation and increases proteasome activity, altering normal proteostasis.

We observed that, in our experimental paradigms, the A53E mutation affects specific steps of the aggregation process of aSyn and different cellular processes, providing novel ideas about the molecular mechanisms involved in synucleinopathies.

**Electronic supplementary material:**

The online version of this article (doi:10.1186/s40478-016-0402-8) contains supplementary material, which is available to authorized users.

## Introduction

Parkinson’s disease (PD) is a highly debilitating and progressive neurodegenerative disorder affecting around seven million people worldwide. PD is typically known as a movement disorder, due to the characteristic motor manifestations associated with the loss of dopaminergic neurons from the *substantia nigra*, although it also affects other areas of the brain. PD and other neurodegenerative disorders, such as demential with Lewy bodies, and multiple system atrophy, are also characterized by the accumulation of aggregated alpha-synuclein (aSyn) in proteinaceous inclusions known as Lewy bodies (LBs) or Lewy neurites [54]. Together, these diseases are known as synucleinopathies [17, 55]. However, it is still unclear whether LBs are themselves toxic or protective [7, 43], with smaller oligomeric species of aSyn being the culprits as recent studies suggest [31, 45, 58, 63]. aSyn is a disordered and abundant neuronal protein whose normal function is still elusive. Familial forms of PD associated with duplication and triplication of the *SNCA* gene [53], along with studies of aSyn overexpression, in cellular and animal models, suggest the protein may acquire a toxic function. The cellular pathologies associated with increased levels and accumulation of aSyn include disruption of vesicular transport [6, 42], mitochondrial dysfunction, impairment of autophagy and proteasome, and oxidative stress [2, 21], suggesting aSyn plays a multitude of roles in the cell, perhaps due to its intrinsically disordered nature. Under physiological conditions, aSyn is considered to be a pre-synaptic protein [37] that associates with vesicles and membranes [11].

According to the “Braak hypothesis”, PD pathology is thought to start from the periphery (gut or nose), and progress until it reaches the brain [4, 5, 49], spreading in a prion-like manner [20, 29, 36]. However, this hypothesis is still controversial, and the molecular mechanisms underlying this phenomenon are not fully understood [25].

The vast majority of PD cases are sporadic but single point mutations in the gene encoding for aSyn (*SNCA*) cause familial forms of the disease [10]. The most recently identified aSyn mutation causes the substitution of alanine at position 53 by a glutamate residue (A53E), identified in a 36 year-old Finnish patient with atypical PD. The patient displayed a dense accumulation of *SNCA* inclusions in the striatum and a severe cortical pathology, affecting both the superficial and deep laminae [3]. In vitro, the A53E mutation was shown to reduce aSyn aggregation and fibril formation without changing the secondary structure content of the protein, when compared to WT aSyn [15]. These data suggest that the negatively charged glutamate residue may affect the folding and, consequently, the aggregation process of the protein.

In our study, we conducted a detailed study of the effects of the A53E mutation on aSyn using a combination of in vitro and cellular models of aSyn oligomerization and aggregation [40, 45]. Our results showed that the A53E mutation modulates aSyn aggregation in vitro and in vivo and impacts on distinct cellular pathways.

Altogether, the study of specific aSyn mutants provides novel insight into the spectrum of functions and cellular pathologies associated, opening novel avenues for the design of therapeutic strategies for PD and other synucleinopathies.

## Materials and methods

### Protein expression and purification

pET21a vectors (Novagen) encoding for WT aSyn and the A53E mutant were transformed into *E. coli* BL21 (DE3) cells. For protein expression, 10 ml overnight culture of transformed cells was used to inoculate 1 L of LB medium with 100 μg/mL ampicillin, which was further incubated at 37 °C and 250 rpm. At an OD_600_ 0.6, protein expression was induced with 1 mM of isopropyl-1-thio-β-D-galactopyranoside (IPTG) for 4 h at 37 °C. Afterwards, the cultures were centrifuged and the cell pellet frozen at −80 °C.

For cell lysis, pellets were resuspended in 15 mL of lysis buffer (50 mM Tris · HCl, 150 mM NaCl, 1 μg/mL Pepstatin A, 20 μg/ml Aprotinin, 1 mM Benzamidine, 1 mM PMSF and 1 mM EDTA) and sonicated on ice. The lysate was boiled for 10 min at 95 °C and soluble and insoluble fractions were separated by centrifugation at 48,384 × *g*, for 15 min. 136 μL/mL of 10% streptomycin sulfate and 228 μL/mL of glacial acetic acid was added to the supernatant. The resulting solution was centrifuged for 5 min at 48,384 × *g,* and the supernatant was collected and precipitated with saturated ammonium sulphate at 4 °C in a ratio 1:1 (v/v) with the supernatant. The pellets were washed with a 1:1 (v/v) solution of ammonium sulphate and water. The pellets were resuspended in 900 μL of ammonium acetate 100 mM and the same volume of 100% ethanol was added to precipitated aSyn.

Purification protocol was as adapted from [59]. Briefly, ethanol precipitated aSyn was resuspended in starting buffer (25 mM Tris · HCl at pH 8.0) and filtered through a Millex-HP filter syringe-driven filter unit (0.45 μm, Millipore). Anion exchange high-performance liquid-chromatography was carried out on an AKTA-FPLC (GE Healthcare). The sample was loaded, bounded to Hi-Trap column (GE Healthcare) and eluted with a NaCl linear gradient of elution buffer (25 mM Tris · HCl at pH 8.0, 1 M NaCl). The fractions containing aSyn were collected and buffer was exchanged for 20 mM ammonium acetate. The identity, and purity of the recombinant proteins was assessed by Mass Spectrometry and SDS-PAGE being higher than 99%.

### Aggregation assays

The protein stocks were prepared by resuspending lyophilized protein in native buffer (10 mM sodium phosphate at pH 7.0) to a concentration of approximately 100 μM. Then, the protein stocks were filtered through 0.22 μm filter unit. The integrity of the protein and the absence of soluble oligomers at the beginning of the reaction was confirmed by gel filtration chromatography in a Superdex 75 10/300 column (GE Healthcare Life Sciences). Three aliquots of 300 μL of aSyn WT and A53E mutant were prepared from the protein stocks, in native buffer to a final concentration of 60 μM. Samples were incubated in an Eppendorf Thermomixer Comfort (Eppendorf, USA) with 0.02% sodium azide at 600 rpm and 37 °C.

### Light scattering spectroscopy

The transition of aSyn from initial soluble monomeric form to aggregated state was determined by measuring light scattering in a Jasco FP-8200 spectrofluorometer (Jasco Inc, MD, USA) with an excitation wavelength of 330 nm and emission range from 320 to 340 nm at 25 °C. Final protein concentration was 10 μM in native buffer. Solutions without protein were used as negative controls. All experiments were carried out in triplicates.

### Thioflavin T binding assay

Th-T binding to amyloid fibrils was recorded using a JASCO FP-8200 spectrofluorometer (Jasco Inc, MD, USA) with an excitation wavelength of 445 nm and emission range from 460 to 600 nm at 25 °C, using a slit width of 5 nm for excitation and emission. The final concentration of Th-T was 25 μM and final protein concentration was 10 μM in native buffer. Solutions without protein were used as negative controls. All experiments were carried out in triplicates.

### Congo red binding assay

CR binding to amyloid fibrils was tested using a Cary-400 Varian spectrophotometer (Varian Inc., Palo Alto, CA, USA) by recording the absorbance spectra from 375 to 675 nm, at 25 °C. A final concentration of 10 μM CR was added to 10 μM protein samples in native buffer. Protein solutions in the presence and absence of CR were used to calculate the differential CR spectra. All experiments were carried out in triplicates.

### Transmission electron microscopy (TEM) assays

For negative staining, incubated samples were diluted in Mili-Q water to 10 μM and 10 μL were then placed on carbon-coated copper grids, and left to stand for 5 min. The grids were washed with distilled water and stained with 2% (w/v) uranyl acetate for 1 min. The morphology of aggregates of WT and A53E aSyn was observed using a JEOL JEM 1400 transmission electron microscope (JEOL, USA) at an accelerating voltage of 120 kV. The width of fibrils for WT and necklace-like structures for A53E was measured using ImageJ (250 measurements). Results are provided as Mean ± SE.

### ATR-FTIR spectroscopy

Attenuated total reflectance Fourier transform infrared spectroscopy (ATR FT-IR) analysis of amyloid fibrils was performed using a Bruker Tensor 27 FTIR Spectrometer (Bruker Optics Inc.) with a Golden Gate MKII ATR accessory. Incubated samples were centrifuged and the insoluble fraction was resuspended in water. Each spectrum consists of 16 independent scans, measured at a spectral resolution of 4 cm^−1^ within the 1800–1500 cm^−1^ range. Second derivatives of the spectra were used to determine the frequencies at which the different spectral components were located. FTIR spectra were fitted to overlapping Gaussian curves using PeakFit package software (Systat Software).

### Aggregation kinetics

Aggregation of aSyn, departing from soluble monomeric form, was monitored by measuring the transition from non-aggregated to aggregated state according to the Th-T fluorescence at 486 nm on a 96-wells microplate reader for 72 h at 37 °C (Victor Microplate reader, Perkin Elmer, USA). The reactions were carried out with 70 μM soluble purified WT and A53E aSyn in native buffer. Experiments were carried out in triplicates.

### Sedimentation assay

aSyn aggregation was measured by sedimentation using centrifugation. The incubated samples of WT and A53E mutant were centrifuged at 48,384 × g for 30 min, and the supernatants were carefully removed. The amount of soluble aSyn was measured by absorbance at 280 nm using aSyn extinction coefficient Ɛ_280_ = 5960 M^−1^/cm^−1^, before and after centrifugation. All measurements were carried out in triplicates.

### Primer design

The primers were designed according with the manufacturer’s instructions (Table 1).

**Table 1:** Primers used to perform site-directed mutagenesis and generate the A53E mutant

A53E forward	5′ GAGTGGTGCATGGTGTGGAAACAGTGGCTGAGAAGAC 3′
A53E reverse	5′ GTCTTCTCAGCCACTGTTTCCACACCATGCACCACTC 3′

### Generation of A53E aSyn constructs for expression in mammalian cells

A53E was inserted in the Venus-BiFC system [45] or SynT [40] by site-directed mutagenesis (QuickChange II Site-Directed Mutagenesis Kit, Agilent Technologies, SC, USA) following the manufacturer’s instructions. All constructions were confirmed by sequencing.

### Cell culture

Human Embryonic Kidney 293 (HEK) cells were grown in Dulbecco’s Modified Eagle Medium (DMEM, Life Technologies- Invitrogen, Carlsbad, CA, USA), and human neuroglioma cells (H4) in Opti-MEM I with Glutamax (Life Technologies- Gibco, Carlsbad, CA, USA), both supplemented with 10% Fetal Bovine Serum Gold (PAA, Cölbe, Germany) and 1% Penicillin-Streptomycin (PAN, Aidenbach, Germany). Cells were grown at 37 °C, with 5% of CO2.

### Cell transfection

#### HEK cells

The day before transfection, 100 000 cells were plated in 12-well plates (Costar, Corning, New York, USA). The cells were transfected with equimolar amounts of the plasmids using Metafectene (Biotex, Munich, Germany) as specified by the manufacturer. After twenty-four hours, the cells were collected or stained for further analysis.

#### H4 cells

Eighty thousand cells were plated in 12-well plates (Costar, Corning, New York, USA). After 24 h, equal amount of SynT and Synphilin-1 were transfected using FuGENE6 Transfection Reagent (Promega, Madison, USA) in a ratio of 1:3 according to the manufacturer’s recommendation. Forty-eight hours after transfection, the cells were processed for different assays.

### Immunocytochemistry

After transfection, cells were fixed with 4% paraformaldehyde at room temperature (RT), followed by a permeabilization with 0.5% Triton X-100 (SigmaAldrich, St. Louis, MO, USA). The cells were blocked in 1.5% normal goat serum (PAA, Cölbe, Germany)/1xPBS (1.37 M NaCl, 27 mM KCl, 101.4 mM Na_2_HPO_4_7^.^H_2_O, 16.7 mM KH_2_PO_4_), and then incubated with primary antibody. Primary antibodies used in this study were: mouse Syn1 (1:1000, BD Transduction Laboratory, New Jersey, USA) or rabbit anti-aSyn (1:1000, Abcam, Boston, USA), anti-Giantin (1:1000, Abcam, Boston, USA), aSyn-S129 1:1000 (Wako Chemicals USA, Inc., Richmond, USA) overnight, and secondary antibody (Alexa Fluor 488 donkey anti-mouse IgG and/or Alexa Fluor 555 goat anti rabbit IgG, (Life Technologies- Invitrogen, Carlsbad, CA, USA)) for 2 h at RT. Cells were finally stained with Hoechst 33258 (Life Technologies- Invitrogen, Carlsbad, CA, USA) (1:5000 in DPBS) for 5 min, and maintained in 1xPBS for imaging.

### Yeast transformation and plasmids

The p426GAL-aSyn-GFP plasmid carries the human gene of aSyn with a C-terminal fusion to GFP, under the regulation of *GAL1* inducible promoter [44]. This plasmid was used to generate aSyn A53E by site directed mutagenesis. The yeast cells W303-1A (*MAT* **a**; *can1-100*; *his3-11*,*15*; *leu2-3,112*; *trp1-1*; *ura3-1*; *ade2-1*) were transformed with the indicated plasmids using lithium acetate standard method [16].

### Yeast growth

For all experiments, an inoculum was prepared to obtain cells in *log* growth phase, using synthetic complete (SC) medium [0.67% (w/v) yeast nitrogen base without amino acids (Difco), 1% (w/v) raffinose and 0.79 g.L^−1^ complete supplement mixture (CSM) (QBiogene)], 200 rpm, 30 °C, as we described [57]. To induce aSyn expression, in liquid medium, cells were grown (OD_600 nm_ 0.2) in SC selective medium 1% (w/v) galactose (aSyn ON) for 7 h at 30 °C, 200 rpm. aSyn cytotoxicity was evaluated by spotting assays. OD_600 nm_ was set to 0.1 ± 0.005 and 1:10 serially dilutions of each sample were prepared [57]. Then, 4 μL of each dilution was spotted in solid SC selective medium containing 2% glucose (aSyn OFF) or 1% (w/v) galactose (aSyn ON) and incubated at 30 °C for 36–42 h. Images were acquired using ChemiDoc Touch (Bio-Rad).

### Fluorescence microscopy

HEK cells expressing the aSyn Venus-BiFC assay were visualized using the Olympus IX81-ZDC microscope system, with a 20× objective. One hundred images were randomly taken out of four independent experiments. Total intensity was measured using the Olympus Scan^R Image Analysis Software.

In order to determine the percentage of yeast cells with aSyn inclusions, cells were grown as described above and GFP fluorescence was visualized with a Zeiss Z2 Widefield Fluorescence microscope. The percentage of cells presenting aSyn inclusions was then determined by counting at least 300 cells for each treatment using ImageJ software.

### Quantification of aSyn inclusions

Cells expressing aSyn were scored according with the presence or absence of inclusion on transfected cells. Three independent experiments were performed, and the results were expressed as the percentage of the total number of transfected cells.

### Thioflavin S staining

Freshly prepared, 0.5% of Thio-S (Sigma-Aldrich, St. Louis, MO, USA) was incubated within the cells for 5 min. The cells were washed three times with 80% ethanol, and maintained in 1xPBS for fluorescence microscopy.

### Quantification of Golgi fragmentation

Golgi morphology was assessed in transfected HEK and H4, and scored into three groups, was we previously published [32] (normal, diffused and fragmented). Three independent experiments were performed.

### Western blot analysis

HEK and H4 cells were lysed with Radio-Immunoprecipitation Assay (RIPA) lysis buffer (50 mM Tris pH 8.0, 0.15 M NaCl, 0.1% SDS, 1% NP40, 0.5% Na-Deoxycholate), 2 mM EDTA and a Protease Inhibitor Cocktail (1 tablet/10 mL) (Roche Diagnostics, Mannheim, Germany). Protein concentration was determined by Bradford assay (BioRad Laboratories, Hercules, CA, USA), and the sample were denaturation for 5 min at 100 °C in protein sample buffer (125 mM of 1 M Tris HCl pH 6.8, 4% SDS 0,5% Bromophenol blue, 4 mM EDTA, 20% Glycerol, 10% b-Mercapto ethanol). The gels were loaded with 80 μg protein, and the samples separated on 12% SDS-polyacrylamide gels. The gel was transferred to a PVDF membrane using a Trans-Blot Turbo transfer system (BioRad), according to the manufacturer’s instructions. Membranes were blocked with 5% (w/v) skim milk (Fluka, Sigma-Aldrich, St. Louis, MO, USA), and incubated with Syn1 (1:1000, BD Biosciences, San Jose, CA, USA), and 1:2000 anti-b-actin (Sigma-Aldrich, St. Louis, MO, USA) overnight at 4 °C. After washing, the membranes were incubated for 1 h with secondary antibody, anti-mouse IgG, or anti-rabbit IgG, horseradish peroxidase labeled secondary antibody (GE Healthcare, Bucks, UK) at 1:10,000. Proteins were detected by ECL chemiluminescent detection system (Millipore, Billerica, MA, USA) in Fusion FX (Vilber Lourmat). The band intensity was estimated using the ImageJ software (NIH, Bethesda, MD, USA) and normalized against b-actin.

For aSyn quantification total yeast protein extraction and western blot was performed following standard procedures as described before [57]. Antibodies used: aSyn (BD Transduction Laboratories, San Jose, CA, USA), pS129-aSyn (Wako Chemicals USA, Inc., Richmond VA, USA) PGK (Life Technologies, PaisleyUK). Triton soluble and insoluble fractions were processed and analyzed as described before [56].

### Native PAGE

For native PAGE, HEK cells were lysed in 1xPBS pH 7.4 with Protease Inhibitor Cocktail tablet and separated in 4–16% gradient Native pre-cast gel (SERVA Electrophoresis GmbH, Heidelberg, Germany). Gels were run according to the manufacturer’s instructions, and transferred as previously described.

### Proteinase K digestion

H4 cells samples were digested with Proteinase K (2.5 μg/mL) (Roth, Carlsbad, Germany) for 1, 3, and 5 min at 37 °C. The enzyme reaction was stopped with protein sample buffer, and the samples were separated in a SDS-page gel, as described above.

### Flow cytometry (FCM)

FCM was performed in a BD FACSCanto II. To analyze cell viability yeast cells transformed with the indicated plasmids were incubated with 5 μg.mL^−1^ PI, for 15 min at 30 °C, 200 rpm and protected from light. Cells were then washed with PBS and used to FCM. A minimum of 10,000 events were collected for each experiment. Results were expressed as median fluorescence intensity (MFI) of a molecule.

### Measurement of 26S Proteasome Catalytic Activity

The chymotrypsin-like activity of the 26S proteasome was determined has previously described [27]. Briefly, after 48 h transfected cells were collected in lysis buffer (50 mM Tris, pH 7.5, 250 mM Sucrose, 5 mM MgCl2, 1 mM DTT, 0.5 mM EDTA, 0.025% Digitonin, 2 mM ATP). The reaction was initiated with 15 μg from the total protein lysates, together with the addition of reaction buffer (50 mM Tris (pH 7.5), 40 mM KCl2, 5 mM MgCl2, 1 mM DTT, 0.5 mM ATP, 100 μM Suc-LLVYAMC) were mix in 100 μl final volume. The fluorescence of AMC (380 nm exCitation and 460 nm emission) was monitored in a microplate fluorometer (Infinite M1000, Tecan) at 37 °C. As control, proteasome was inhibited with 20 μM MG132 (Sigma, Hamburg, Germany) prior to the measurements.

### Statistical analyses

Data were analyzed using GraphPad Prism 6 (San Diego California, USA) software and were expressed as the mean + − SD. Statistical differences from WT aSyn were calculated using unpaired Student *t*-test and one-way ANOVA with post-hoc Tukey’s test. Significance was assessed for, where * corresponds to *p* < 0.05, ** corresponds to *p* < 0.01 and *** corresponds to *p* < 0.001.

## Results

### A53E mutant forms protofibrils by reducing aSyn fibrilization

**Fig. 1:**
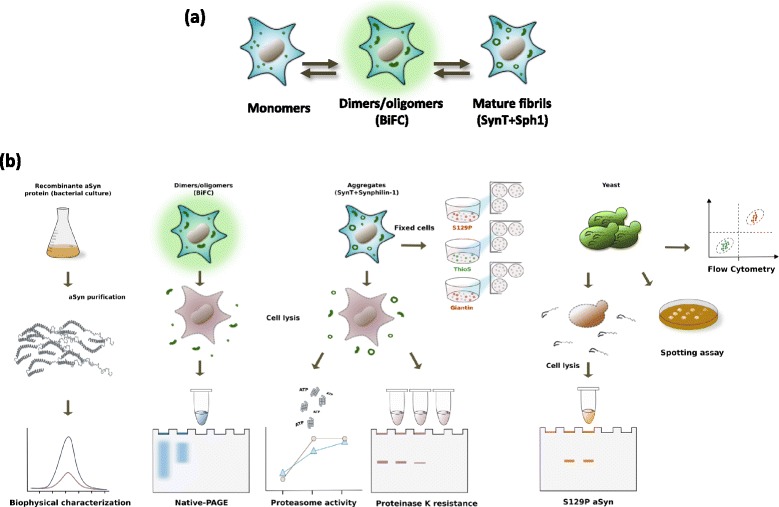
Aggregation process and study design. **a** aSyn aggregation process in cell models. **b** Experimental design used in the study. In vitro studies and studies in cell models were used to assess the effect of the A53E mutation on aSyn

To understand the effect of the A53E substitution in the aggregation process of aSyn (Fig. 1a), we assembled a pipeline of both in in vitro and cellular studies, in human and yeast cells (Fig. 1b), and conducted a battery of experiments to characterize the behavior of this recently identified aSyn mutant form.

**Fig. 2:**
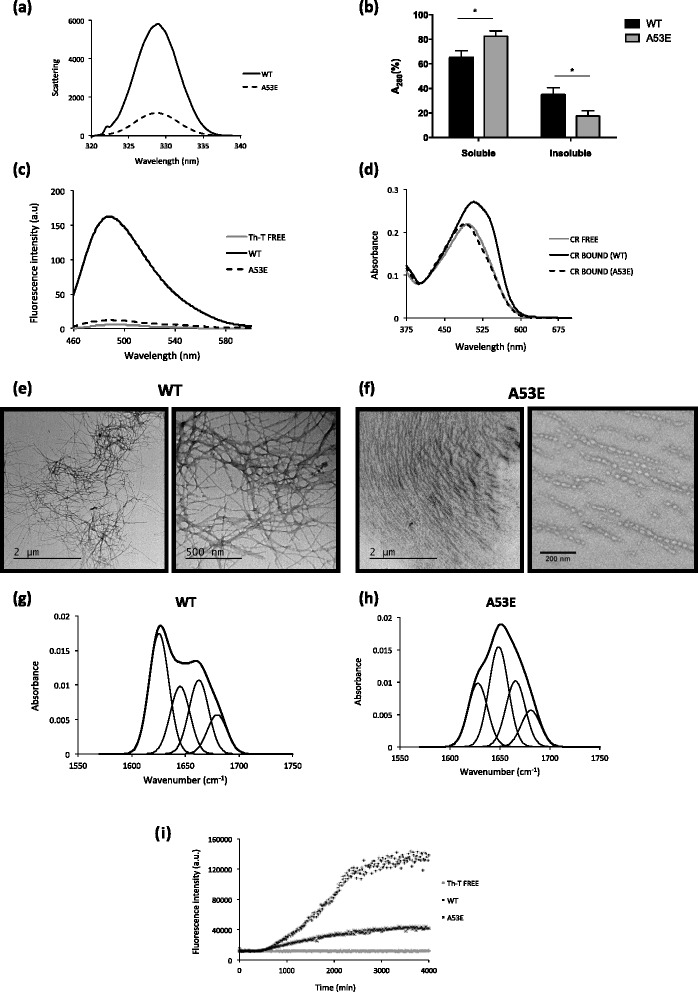
Aggregation properties of WT and A53E aSyn variants. aSyn WT and A53E mutant, prepared at 60 μM in 10 mM sodium phosphate, pH 7.0, were incubated for 2 weeks under agitation at 37 °C. **a** Static light scattering of 10 μM aSyn in 10 mM sodium phosphate, WT (*solid line*) and A53E mutant (*dashed line*). **b** Distribution of aSyn between the soluble and insoluble fractions. **c** Fluorescence emission spectra of Th-T upon incubation with 10 μM aSyn WT (*solid line*) and A53E mutant (*dashed line*). Free Th-T emission spectrum is represented in grey. **d** CR absorbance spectra in the presence of 10 μM aSyn WT (solid line) and A53E mutant (*dashed line*). Free CR absorbance spectrum is represented in grey. **f**-**g** Morphology of WT and A53E aSyn aggregates TEM micrographs. Negatively stained aggregates formed by aSyn WT (left panel) and A53E mutant (right panel) incubated for two weeks. **h**-**i** Secondary structure of WT and A53E aSyn aggregates. Secondary structure content of the aSyn WT and A53E mutant after two weeks incubation. ATR-FTIR absorbance spectra in the amide I region was acquired (thick line) and the fitted individual bands after Gaussian deconvolution are shown (*thin lines*). **i** Aggregation kinetics of WT and A53E aSyn. Aggregation kinetics of aSyn were monitored by following the changes in relative ThioT fluorescence emission. Concentration of protein was 70 μM WT aSyn (crosses) and A53E mutant (*dots*) in a final volume of 150 μL. The evolution of Th-T fluorescence in the absence of protein is represented in *grey*, *n* = 3

We started to compare the in vitro aggregation properties of WT and A53E aSyn using synchronous light scattering, sedimentation and binding to amyloid-binding dyes. For these assays, the soluble forms of both proteins were incubated at 60 μM under agitation at 37 °C for two weeks. The light scattering signal was 6-fold higher for WT aSyn than for the A53E mutant, suggesting differences in aggregation process (Fig. 2a). Using spectrophotometry, we quantified the levels of aggregated protein in both solutions after separating aSyn in two fractions (soluble and insoluble), by centrifugation. The amount of protein in the insoluble fraction in the WT aSyn preparation was two times higher than with A53E (*p < 0.05, Fig. 2b).

The presence of amyloid fibrils can be detected in vitro using Thioflavin T (Th-T), a dye that specifically binds to amyloid fibrils [35, 51]. In agreement with light scattering and sedimentation data, we found that the Th-T fluorescence signal was 10 times lower for A53E than for WT aSyn (Fig. 2c). The presence of amyloid fibrils can be further detected by monitoring the increase of the absorbance of Congo Red (CR) and the red shift of the dye absorbance maximum [28]. The binding of A53E to CR was negligible, since the peak of absorbance in the presence of A53E aSyn was similar to that of free CR (Fig. 2d). In contrast, we observed a dramatic spectral change in CR for WT aSyn (Fig. 2d). To confirm the different amyloidogenic propensities of WT and A53E aSyn, we analyzed the morphological features of the aggregates formed using transmission electron microscopy (TEM). Although we detected the presence of higher order complexes in both preparations, their size and morphology was different. For WT aSyn, we observed the typical long and unbranched amyloid fibrils (Fig. 2e), 11.6 ± 0.4 nm with a width of 11.6 ± 0.4 nm (Fig. 2e). In contrast, the structures formed by A53E aSyn exhibited a protofibrillar appearance, with small round oligomeric structures that seemed to be linked in a necklace fashion, with a width of 28.5 ± 0.7 nm (Fig. 2f-g).

To assess the secondary structure content of the assemblies formed by WT and A53E aSyn, we analyzed the amide I region of the FTIR spectrum (1700–1600 cm^−1^). This region of the spectrum corresponds to the absorption of the carbonyl peptide bond of the main amino acid chain of the protein, and is a sensitive marker of the protein secondary structure. After deconvolution of the FTIR spectra of the aSyn solutions, we were able to assign the individual secondary structure elements and their relative contribution to the main absorbance signal at the end of the aggregation reaction (Fig. 2g and h and Table 2). The absorbance spectra were radically different for WT and A53E aSyn. While the spectrum of WT aSyn was dominated by a peak at 1625 cm^−1^, attributable to the presence of amyloid-like inter-molecular β–sheet structure (Fig. 2g), the spectrum of the A53E mutant was dominated by a peak at 1649 cm^−1^ corresponding to disordered/random coil conformation (Fig. 2h).

**Table 2:** Assignment of secondary structure components of aSyn variants in the amide I region of the FTIR spectra

	WT			A53E		
	Band (cm^−1^)	Area (%)	Structure	Band (cm^−1^)	Area (%)	Structure
1	1625	40	β -sheet (inter)	1628	24	β -sheet (inter)
2	1645	22		1649	38	
3	1663	25	Loop/β-turn/bend/α-helix	1666	25	Loop/β-turn/bend/α-helix
4	1680	13	β-turn	1681	14	β-turn

Next, we monitored how the mutation impacted on the aggregation kinetics of aSyn by continuously monitoring the changes in Th-T binding over time for WT and A53E variants. The kinetics of amyloid fibril formation usually follows a sigmoidal curve that reflects a nucleation-dependent growth mechanism. The aggregation of both proteins followed this pattern, with an apparent lag phase of 8 h (Fig. 2i). After this lag phase, the two aggregation reactions diverged significantly, with an exponential increase for WT aSyn that plateaued at around 55 h, and a steady and much slower increase for A53E aSyn, reaching a 3.5-times lower fluorescence intensity. Altogether, our data demonstrates that the A53E mutation reduces aSyn amyloid formation in vitro.

### The A53E mutation decreases aSyn oligomerization in cellular models

**Fig. 3:**
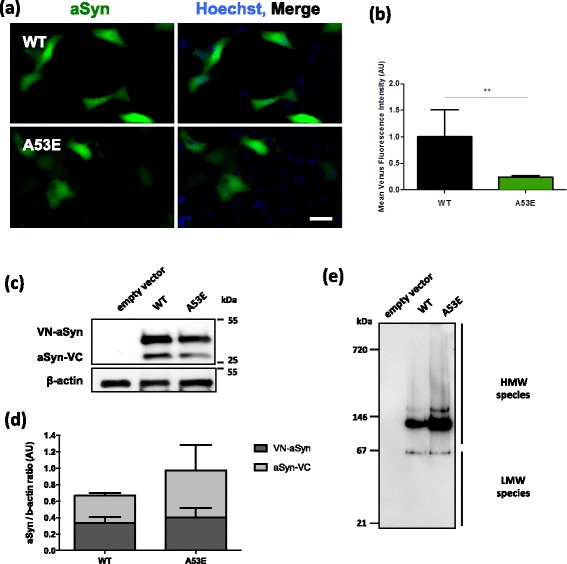
A53E reduces aSyn oligomerization. **a** Fluorescent cells, expressing VN-aSyn and aSyn-VC constructs, as a result of the aSyn interaction. Scale bar: 30 μm. **b** Mean fluorescence intensity of cells were assessed 24 h post-transfection using an Olympus IX81-ZDC microscope. For each condition, 100 pictures were acquire in 4 independent experiments were conducted. Student’s *t* test (**p < 0.01). **c**-**d** aSyn protein levels were assessed by immunoblot analysis and were found to similar between WT aSyn and the A53E mutant. *n* = 3. **e** Native-PAGE gel showed that the A53E mutant forms high molecular weight species similar to WT aSyn

We next investigated the effects of the A53E mutation on the behavior of aSyn in the context of living human cell models. First, we used the Bimolecular Fluorescence Complementation (BiFC) assay to monitor aSyn oligomerization, as we previously described [45]. Briefly, non-fluorescence Venus fragments are fused to either the N- or C-terminus of aSyn and, upon dimerization/oligomerization of the protein, the fluorophore is reconstituted resulting in fluorescence signal. While this assay involves the tagging of aSyn with fragments of fluorescent proteins, it constitutes a powerful paradigm to assess aSyn oligomerization. We observed that A53E aSyn, similarly to WT, formed dimers/oligomers (Fig. 3a). However, the fluorescence signal was lower than that observed with WT aSyn, suggesting differences in the dimerization/oligomerization process (**p < 0.01) (Fig. 3b), since the levels of expression of WT and A53E aSyn were identical (Fig. 3c and d, Additional file 1: Figure S2.1 and Additional file 1: Figure S2.2). We also found that the A53E mutant produced high molecular weight species, similar to WT aSyn (Fig. 3e). Thus, we refer to the species formed as oligomers, for simplicity.

### The A53E mutation alters the biochemical properties of aSyn inclusions

**Fig. 4:**
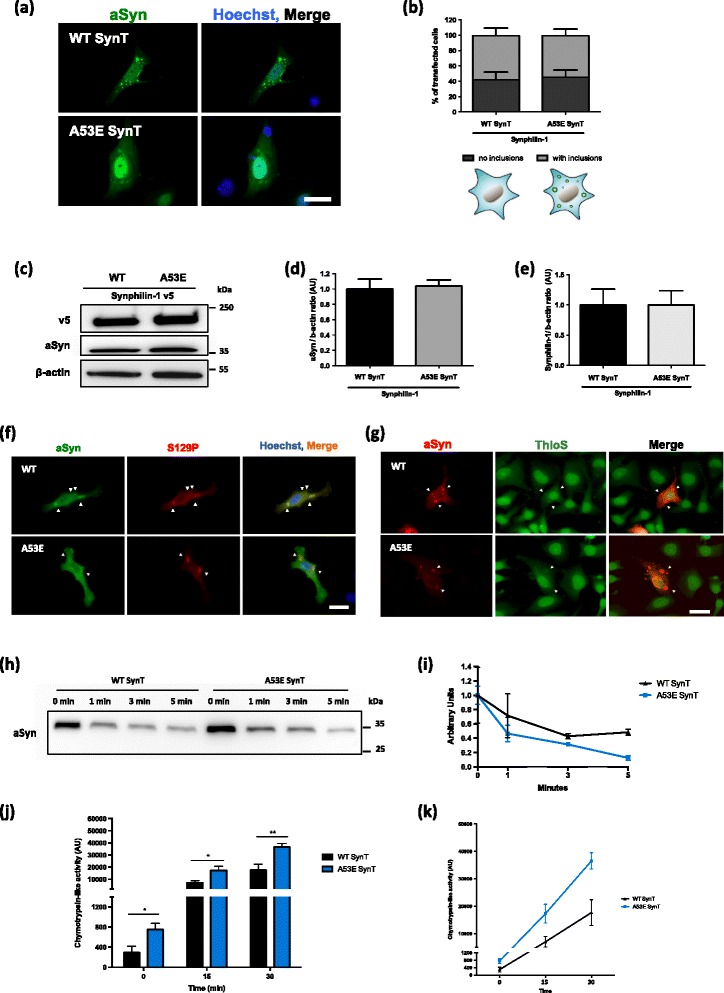
A53E does not change the inclusion pattern. **a**-**b** At least 50 cells *per* condition were classified according to the pattern formed. We observed that A53E did not change the number of inclusions per cells. *n* = 3. Scale bar: 30 μm. **c**-**e** Immunoblot analysis of the aSyn and Synphilin-1 levels showed no significant differences in expression of WT or A53E aSyn. *n* = 3 (**f**) Inclusions formed by WT and A53E are positive for pS129. Positive inclusions are indicated with *white arrows*. Scale bar: 30 μm. **g** Inclusions were stained with Th-S and analyzed *via* fluorescence microscopy. As indicated with *arrow* heads, we observed that some inclusions displayed amyloid-like properties, by staining positive with Th-S. Scale bar: 30 μm. **h**-**i** WT and A53E aSyn protein lysates were digested with PD for different times (1, 3 and 5 min). After normalization of the values to the undigested condition, we observed that A53E inclusions are less resistant to PK-digestion. *n* = 2. **j**-**k** 48 h post-transfection the cells were collected and we assessed the activity of the proteasome. We observed that cells expressing A53E mutant increased proteolytic activity of the proteasome in comparison with WT. *n* = 3. Student’s *t* test (*p < 0.05, **p < 0.01)

Next, we investigated if the later stages of the aSyn aggregation process were altered by the A53E mutation. For that, we used a well-established cell-based aggregation model that consists in the co-expression of SynT (C-terminally modified aSyn) and Synphilin-1 [40], since expression of aSyn alone does not result in inclusion formation. In this aSyn aggregation model, aSyn inclusions are readily detected by immunocytochemistry using antibodies against aSyn, allowing the characterization of different types of inclusions [32], and screening modulators of aSyn aggregation [41]. 48 h after transfection, we analyzed inclusion formation in the cells (Fig. 4a), and observed that the A53E mutation did not alter inclusion formation when compared to WT aSyn (Fig. 4b). Again, no differences in the levels of aSyn or Synphilin-1 were detected between WT and A53E mutant aSyn (Fig. 4c-e).

aSyn is phosphorylated on Serine 129 (pS129) in LBs found in the brains of PD patients, linking this posttranslational modification with disease [14, 52]. We previously showed that specific aSyn mutations, such as the E46K, can alter pS129 on aSyn [39]. Thus we investigated the effect of the A53E mutation on S129 phosphorylation. We found that both WT and A53E aSyn inclusions were positive for pS129 (Fig. 4f).

To further investigate the biochemical nature of the inclusions formed by the A53E aSyn mutant, we stained the cells with Thioflavin S (Th-S), a dye that binds to β-sheet rich amyloid structures [33]. We observed that the larger inclusions formed by WT and A53E aSyn stained positive for Th-S (Fig. 4g). In addition, we also used proteinase K (PK) resistance as a marker of aggregate formation, as protein inclusions tend to be more resistance to PK digestion. Interestingly, we found that the A53E mutant was less resistant when compared to WT aSyn (Fig. 4h and i), suggesting the inclusions formed by the A53E mutant have a less-compact nature than those formed by the WT protein.

The ubiquitin-proteasome system (UPS) is the major non-lysosomal pathway for selective protein degradation. In cell models, it has been shown that aSyn accumulation can affect the activity of the UPS system [61]. In our experimental conditions, we observed that cells expressing A53E aSyn mutant display increased proteolytic activity of the proteasome (Fig. 4j and k).

### aSyn aggregation leads to Golgi fragmentation

**Fig. 5:**
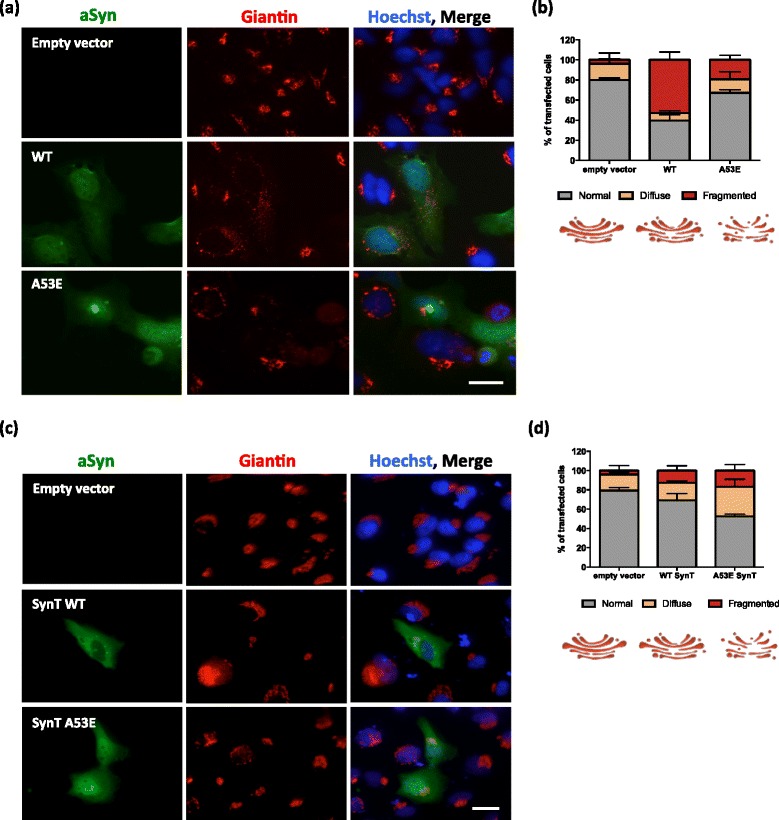
Golgi morphology in the oligomerization and aggregation models. **a**-**b** Representative pictures of transfected cells with the BiFC system. We analyzed and categorized transfected cells in different categories. Scale bar: 30 μm. **b** WT aSyn resulted in an increased percentage of cells with fragmented Golgi morphology when compared with the empty vector and with cells expressing the A53E mutant. **c**-**d** Representative pictures of transfected cells with SynT + Synphilin-1. Scale bar: 30 μm. **d** In the presence of A53E SynT, the Golgi is more diffuse when compared with the control and with WT SynT. *n* = 3

aSyn induces several cellular pathologies that have been documented over the years and are routinely used to assess the effect of specific mutations or genetic interactors [60]. One particular type of cellular pathology associated with aSyn toxicity is the fragmentation of the Golgi apparatus [13]. This is also evident in other neurodegenerative diseases [18], suggesting it might be a more general response to the proteotoxicity associated with protein misfolding and aggregation. To assess whether expression of A53E mutant aSyn affected the integrity of the Golgi, we analyzed the morphology of this organelle in both the aSyn oligomerization and aggregation models (Fig. 5a and c). We classified the morphology of the Golgi as normal, diffuse and fragmented, as we previously described [32].

In the oligomerization model, we observed that WT aSyn reduced the percentage of cells exhibiting normal Golgi morphology (~40%, ***p < 0.001 Fig. 5a and b and Additional file 1: Figure S3.1–3.3), as we previously reported [32]. However, in cells expressing the A53E mutant the effects were not as pronounced as with WT, and the phenotype was more similar to that of cells carrying an empty vector (~70% and 80% of the transfected cells displayed normal Golgi morphology for A53E, and empty vector, respectively) (*p < 0.05) (Fig. 5b and Additional file 1: Figure S3.1).

In the aSyn aggregation model, we observed the opposite effect. Around 50% of the cells expressing the A53E SynT displayed normal Golgi morphology whereas around 70% of the cells expressing WT SynT displayed normal Golgi (Fig. 5c and d and Additional file 1: Figure S2.4–2.6; ***p < 0.001 and *p < 0.01 for empty vector, and WT, respectively). This suggests that the aggregation of A53E aSyn induces Golgi alterations.

### A53E aSyn behaves identically to WT aSyn in yeast cells

**Fig. 6:**
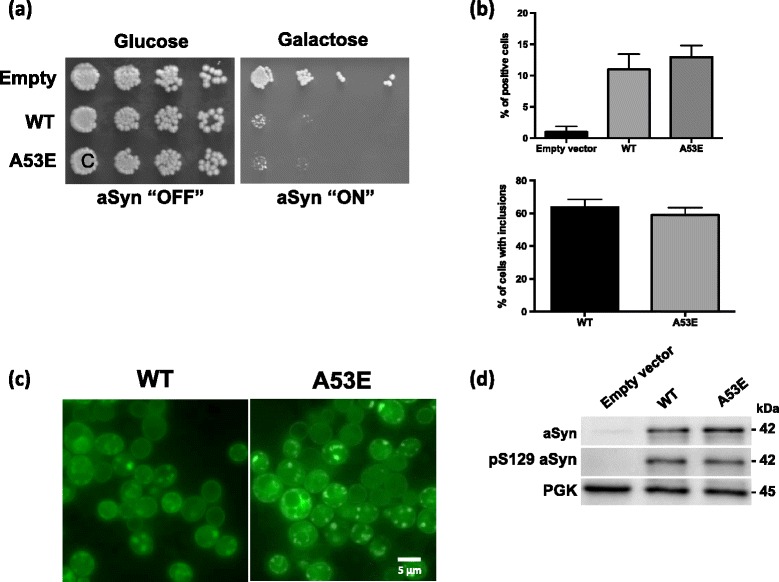
Phenotypic characterization of yeast cells expressing the A53E aSyn mutant. **a** Cytotoxicity of WT and A53E aSyn in yeast cells compared to the empty vector, assessed by spotting assay. Photos were taken 3 days after incubation at 30 °C. **b** Frequency of PI positive cells assessed by flow cytometry, after 7 h of induction of expression of WT and A53E aSyn. **c** Fluorescence microscopy visualization (*left panel*) and percentage of cells with WT and A53E aSyn inclusions (*right panel*). **d** Expression levels of WT and A53E aSyn-GFP in yeast cells assessed by western blot analysis of total protein extracts. Results shown are from one representative experiment from at least three independent experiments. Values represent the mean ± SD of at least three independent measurements

Yeast cells have been extremely useful to assess cellular pathologies associated with the expression of aSyn. Thus, in order to assess if the A53E mutation alters the cytotoxicity and aggregation of aSyn, we expressed this mutant in *S. cerevisiae* and monitored phenotypes previously established [44]. We expressed WT or mutant A53E aSyn fused to eGFP using multi-copy (2 μ) plasmids and under the regulation of a galactose-inducible promoter (*GAL1*). aSyn cytotoxicity was first evaluated by a spotting assay. The growth of the cells expressing A53E aSyn was compared to that of cells expressing WT aSyn (Fig. 6a). As described before, expression of WT aSyn is toxic and results in reduced cell growth (Fig. 6a). We found that cells expressing A53E aSyn display a similar phenotype, suggesting that this familial mutation does not significantly affect aSyn toxicity in yeast (Fig. 6a). To further dissect the cytotoxicity of the A53E mutant, we performed propidium iodide (PI) staining and flow cytometry analysis, a readout of plasma membrane integrity (Fig. 6b). We observed that, 7 h after induction of aSyn expression, no significant differences were observed between cells expressing either WT or the A53E aSyn (Fig. 6b).

Next, we evaluated the subcellular distribution of the A53E mutant aSyn, using fluorescence microscopy. 7 h after induction of aSyn expression we assessed inclusion formation in the cells (Fig. 6c). The expression of A53E aSyn resulted in the formation of cytoplasmic inclusions that looked similar to those formed in cells expressing aSyn WT (Fig. 6c). In addition, no significant differences were observed in the percentage of cells with inclusions between WT and the A53E (Fig. 6c). We also evaluated the levels of aSyn expression by immunoblot analyses and found that both proteins were expressed at similar levels (Fig. 6d). The levels of phosphorylation on serine 129 were also indistinguishable between WT and A53E mutant aSyn (Fig. 6d).

## Discussion

aSyn plays a major role in the pathological processes involved in neurodegenerative diseases, like PD or Dementia with Lewy Bodies [26]. When overexpressed in cells, to mimic familial forms of PD associated with multiplications of the aSyn gene, aSyn can promote cytotoxicity and impair vital processes, thereby contributing to cell death [9, 38, 44]. However, the precise molecular mechanisms underlying aSyn toxicity are still unclear, compromising our ability to intervene therapeutically. Both mutations and multiplications of the *SNCA* gene cause familial forms of PD [8, 22, 64]. Currently, six missense mutations in aSyn have been associated with autosomal dominant forms of parkinsonism (A30P, E46K, H50Q, G51D, A53E, A53T) [1, 30, 34, 46–48, 65]. Of these, the A53E was the last one to be identified and, therefore, has been less investigated.

In this study, we aimed to investigate the effect of the substitution of the alanine at position 53 by a glutamic acid residue that introduces an additional negative charge in the protein. For this purpose, we used in vitro techniques to characterize the biophysical effects of the mutation, and exploited cell-based models to assess the effects of the expression of the A53E mutant on the distribution, aggregation, and toxicity of the protein.

In vitro*,* we observed that A53E attenuates aSyn aggregation and reduces amyloid fibril formation when compared to WT aSyn. In fact, the formation of amyloid structures by the A53E mutant is marginal, since A53E is unable to bind CR. Overall, these observations are in line with a previous report showing that the presence of a negatively-charged residue can reduce the intrinsic aggregation propensity of aSyn [15]. Nevertheless, because the change in net charge in aSyn is small, −9 and −10 for the WT and A53E proteins, respectively, the effect this mutation has on aggregation has more likely a local origin. We used the Amylpred2 consensus aggregation predictor to analyze if the A53E mutation might have an impact in the intrinsic aggregation propensity of the aSyn sequence. Amylpred2 identifies a hot spot of aggregation corresponding to the 49–55 sequence stretch (VHGVATV), including Ala53. The A53E mutation shortens the aggregation region now including only residues 49–53 (VHGVE). The AGGRESCAN algorithm enables the comparison of the aggregation propensity of the two regions, showing that it is 2.1 lower for A53E than for WT aSyn.

In the context of a cell, the behavior of a protein is subjected not only to the crowded environment but also to the action of various protein quality control mechanisms. In human cells, we observed that the A53E mutation reduces aSyn oligomerization without changing the aggregation pattern. Interestingly, the inclusions formed by A53E aSyn are more sensitive to PK digestion than those formed by WT aSyn, suggesting that the inclusions formed are less compact or, possibly, more immature. The negative charge introduced by the glutamate residue can perhaps alter the intermolecular interactions between aSyn molecules, and prevent the formation of tighter inclusions. This may also correlate with the increase in proteasome activity that we observed, since this is an important degradation system to eliminate soluble proteins and smaller assemblies that are not degraded by autophagy.

In our previous studies, we did not observe major differences when comparing another PD-associated aSyn mutant at position 53 (A53T) with WT protein. We found that the aggregation of A53T aSyn was identical to that of WT aSyn [32], suggesting again that the change in charge at position 53 may influence the initial steps of the aggregation process (dimerization/oligomerization) of aSyn, and not the later steps that culminate with the formation of the mature inclusions. Also, the differences might result by difference spatial sequestration of aSyn. Misfolded proteins can be targetted to specific compartments, like aggresomes, to facilitate their degradation [23, 24, 62]. These compartments rely on filament network proteins like vimentin, actin, and tubulin, or proteins that can assist in the transportation of misfolded proteins through the microtubule network, such as p62 (24843142, 12093283, 14623963, 24086678). For example, Tubulin Polymerization Promoting Protein (TPPP/p25), belongs to the microtubule network, and associates with aSyn in pathological conditions, possibly affecting aSyn aggregation (17123092).

The Golgi apparatus plays a determinant role in the intracellular flow of several endogenous proteins and exogenous macromolecules, regulating the trafficking to their final destination inside or outside cells [12]. Fragmentation of the Golgi apparatus is a characteristic feature in several neurodegenerative diseases, including PD [13, 18, 19]. Interestingly, in our cell-based aggregation models, we observed a striking loss of the typical Golgi morphology in cells expressing A53E aSyn. Previous studies about the effect of this recently identified PD mutation in aSyn showed that A53E is as toxic as WT aSyn [15, 50]. While this was also the trend we observed in our cellular models, our findings suggest that the A53E mutant may cause specific alterations in cell physiology that are still not understood and demand additional investigation.

## Conclusions

Overall, our study demonstrates that the A53E aSyn mutation influences the ability of aSyn to aggregate in vitro and in vivo, and that it may induce different cellular pathologies that should be further investigated in vivo. Ultimately, a deeper understanding of the effect of aSyn mutations on the behavior of the protein will enable the design of novel models to assess the potential value of future therapeutic strategies for PD and other synucleinopathies.

## Additional file

Additional file 1: Figure S1.1. Biochemical characterization of WT and A53E recombinant aSyn. Analysis of purified WT and A53E aSyn variants by SDS-PAGE (18%) stained with Coomassie Brilliant Blue. **S1.2.** MALDI-TOF mass spectrometry analysis of purified WT (up) and A53E (down) aSyn variants. A MW of 14462.98 Da was obtained for WT (theoretical of 14460.16 da) and of 14519.47 Da for A53E (theoretical of 14518.19 Da) aSyn variants. The peaks of 7231.82 and 7260.97 Da correspond to the M^+2^ ions of WT and A53E aSyn, respectively. **Figure S2.1 and S2.2.** Immunoblot quantifications. Levels of VN-aSyn (S2.1) and aSyn-VC (S2.1). *n*=3. **Figure S3.1-S3.3.** Morphological analysis of Golgi apparatus in the aSyn BiFC system. The morphology of the Golgi was analyzed as normal (S3.1), diffuse (S3.2) and fragmented (S3.3). One-way ANOVA with post-hoc Tukey’s test (**p*<0.05, ***p*<0.01, ****p*<0.001). *n*=3. **Figure S3.4-3.6** Morphological analysis of Golgi apparatus in the aSyn aggregation model. Transfected cells were analyzed according to the morphology of the Golgi: normal (S3.4), diffuse (S3.5) and fragmented (S3.6) One-way ANOVA with post-hoc Tukey’s test (**p*<0.05, ***p*<0.01, ****p*<0.001). *n*=3. (PDF 389 kb)

## Abbreviations

A53E: Substitution of alanine at position 53 by a glutamate residue
aSyn: alpha-synuclein
ATR FT-IR: Attenuated total reflectance Fourier transform infrared spectroscopy
BiFC: Bimolecular fluorescence complementation assay
CR: Congo red
H4: Human Neuroglioma cells
HEK: Human embryonic kidney cells
IPTG: isopropyl-1-thio-β-D-galactopyranoside
LBs: Lewy bodies
PD: Parkinson’s disease
PI: Propidium iodide
PK: Proteinase K
RT: Room temperature
SC: synthetic complete medium
TEM: Transmission electron microscopy
Th-S: Thioflavin S
Th-T: Thioflavin T
UPS: Ubiquitin-proteasome system

## References

[1] Appel-Cresswell S, Vilarino-Guell C, Encarnacion M, Sherman H, Yu I, Shah B, Weir D, Thompson C, Szu-Tu C, Trinh J (2013). Alpha-synuclein p.H50Q, a novel pathogenic mutation for Parkinson’s disease. Mov Disord.

[2] Auluck PK, Caraveo G, Lindquist S (2010). alpha-Synuclein: membrane interactions and toxicity in Parkinson’s disease. Annu Rev Cell Dev Biol.

[3] Barona-Lleo L, Zulueta-Santos C, Murie-Fernandez M, Perez-Fernandez N (2014). Recent onset disequilibrium mimicking acute vestibulopathy in early multiple sclerosis. Am J Otolaryngol.

[4] Braak H, Del Tredici K, Rub U, de Vos RA, Jansen Steur EN, Braak E (2003). Staging of brain pathology related to sporadic Parkinson’s disease. Neurobiol Aging.

[5] Braak H, Ghebremedhin E, Rub U, Bratzke H, Del Tredici K (2004). Stages in the development of Parkinson’s disease-related pathology. Cell Tissue Res.

[6] Cai H, Reinisch K, Ferro-Novick S (2007). Coats, tethers, Rabs, and SNAREs work together to mediate the intracellular destination of a transport vesicle. Dev Cell.

[7] Chandra S, Gallardo G, Fernandez-Chacon R, Schluter OM, Sudhof TC (2005). Alpha-synuclein cooperates with CSPalpha in preventing neurodegeneration. Cell.

[8] Chartier-Harlin MC, Dachsel JC, Vilarino-Guell C, Lincoln SJ, Lepretre F, Hulihan MM, Kachergus J, Milnerwood AJ, Tapia L, Song MS (2011). Translation initiator EIF4G1 mutations in familial Parkinson disease. Am J Hum Genet.

[9] Cooper AA, Gitler AD, Cashikar A, Haynes CM, Hill KJ, Bhullar B, Liu K, Xu K, Strathearn KE, Liu F (2006). Alpha-synuclein blocks ER-Golgi traffic and Rab1 rescues neuron loss in Parkinson’s models. Science.

[10] Coppede F (2012). Genetics and epigenetics of Parkinson’s disease. Sci World J.

[11] Dikiy I, Eliezer D (2014). N-terminal acetylation stabilizes N-terminal helicity in lipid- and micelle-bound alpha-synuclein and increases its affinity for physiological membranes. J Biol Chem.

[12] Fan J, Hu Z, Zeng L, Lu W, Tang X, Zhang J, Li T (2008). Golgi apparatus and neurodegenerative diseases. Int J Dev Neurosci.

[13] Fujita Y, Ohama E, Takatama M, Al-Sarraj S, Okamoto K (2006). Fragmentation of Golgi apparatus of nigral neurons with alpha-synuclein-positive inclusions in patients with Parkinson’s disease. Acta Neuropathol.

[14] Fujiwara H, Hasegawa M, Dohmae N, Kawashima A, Masliah E, Goldberg MS, Shen J, Takio K, Iwatsubo T (2002). alpha-Synuclein is phosphorylated in synucleinopathy lesions. Nat Cell Biol.

[15] Ghosh D, Sahay S, Ranjan P, Salot S, Mohite GM, Singh PK, Dwivedi S, Carvalho E, Banerjee R, Kumar A, Maji SK (2014). The newly discovered Parkinson’s disease associated Finnish mutation (A53E) attenuates alpha-synuclein aggregation and membrane binding. Biochemistry.

[16] Gietz D, St Jean A, Woods RA, Schiestl RH (1992). Improved method for high efficiency transformation of intact yeast cells. Nucleic Acids Res.

[17] Goedert M, Spillantini MG, Del Tredici K, Braak H (2013). 100 years of Lewy pathology. Nat Rev Neurol.

[18] Gonatas NK, Stieber A, Gonatas JO (2006). Fragmentation of the Golgi apparatus in neurodegenerative diseases and cell death. J Neurol Sci.

[19] Gosavi N, Lee HJ, Lee JS, Patel S, Lee SJ (2002). Golgi fragmentation occurs in the cells with prefibrillar alpha-synuclein aggregates and precedes the formation of fibrillar inclusion. J Biol Chem.

[20] Hardy J (2005). Expression of normal sequence pathogenic proteins for neurodegenerative disease contributes to disease risk: ‘permissive templating’ as a general mechanism underlying neurodegeneration. Biochem Soc Trans.

[21] Hernandez-Vargas R, Fonseca-Ornelas L, Lopez-Gonzalez I, Riesgo-Escovar J, Zurita M, Reynaud E (2011). Synphilin suppresses alpha-synuclein neurotoxicity in a Parkinson’s disease Drosophila model. Genesis.

[22] Ibanez P, Bonnet AM, Debarges B, Lohmann E, Tison F, Pollak P, Agid Y, Durr A, Brice A (2004). Causal relation between alpha-synuclein gene duplication and familial Parkinson’s disease. Lancet.

[23] Johnston JA, Ward CL, Kopito RR (1998). Aggresomes: a cellular response to misfolded proteins. J Cell Biol.

[24] Kaganovich D, Kopito R, Frydman J (2008). Misfolded proteins partition between two distinct quality control compartments. Nature.

[25] Kannarkat GT, Boss JM, Tansey MG (2013). The role of innate and adaptive immunity in Parkinson’s disease. J Park Dis.

[26] Kim WS, Kagedal K, Halliday GM (2014). Alpha-synuclein biology in Lewy body diseases. Alzheimers Res Ther.

[27] Kisselev AF, Goldberg AL (2005). Monitoring activity and inhibition of 26S proteasomes with fluorogenic peptide substrates. Methods Enzymol.

[28] Klunk WE, Pettegrew JW, Abraham DJ (1989). Quantitative evaluation of congo red binding to amyloid-like proteins with a beta-pleated sheet conformation. J Histochem Cytochem.

[29] Kordower JH, Brundin P (2009). Propagation of host disease to grafted neurons: accumulating evidence. Exp Neurol.

[30] Kruger R, Kuhn W, Muller T, Woitalla D, Graeber M, Kosel S, Przuntek H, Epplen JT, Schols L, Riess O (1998). Ala30Pro mutation in the gene encoding alpha-synuclein in Parkinson’s disease. Nat Genet.

[31] Lashuel HA, Overk CR, Oueslati A, Masliah E (2013). The many faces of alpha-synuclein: from structure and toxicity to therapeutic target. Nat Rev Neurosci.

[32] Lazaro DF, Rodrigues EF, Langohr R, Shahpasandzadeh H, Ribeiro T, Guerreiro P, Gerhardt E, Krohnert K, Klucken J, Pereira MD (2014). Systematic comparison of the effects of alpha-synuclein mutations on its oligomerization and aggregation. PLoS Genet.

[33] Lee HJ, Lee SJ (2002). Characterization of cytoplasmic alpha-synuclein aggregates. Fibril formation is tightly linked to the inclusion-forming process in cells. J Biol Chem.

[34] Lesage S, Anheim M, Letournel F, Bousset L, Honore A, Rozas N, Pieri L, Madiona K, Durr A, Melki R (2013). G51D alpha-synuclein mutation causes a novel parkinsonian-pyramidal syndrome. Ann Neurol.

[35] LeVine H (1993). Thioflavine T interaction with synthetic Alzheimer’s disease beta-amyloid peptides: detection of amyloid aggregation in solution. Protein Sci.

[36] Li JY, Englund E, Holton JL, Soulet D, Hagell P, Lees AJ, Lashley T, Quinn NP, Rehncrona S, Bjorklund A (2008). Lewy bodies in grafted neurons in subjects with Parkinson’s disease suggest host-to-graft disease propagation. Nat Med.

[37] Maroteaux L, Campanelli JT, Scheller RH (1988). Synuclein: a neuron-specific protein localized to the nucleus and presynaptic nerve terminal. J Neurosci.

[38] Martin LJ, Pan Y, Price AC, Sterling W, Copeland NG, Jenkins NA, Price DL, Lee MK (2006). Parkinson’s disease alpha-synuclein transgenic mice develop neuronal mitochondrial degeneration and cell death. J Neurosci.

[39] Mbefo MK, Fares MB, Paleologou K, Oueslati A, Yin G, Tenreiro S, Pinto M, Outeiro T, Zweckstetter M, Masliah E, Lashuel HA (2015). Parkinson disease mutant E46K enhances alpha-synuclein phosphorylation in mammalian cell lines, in yeast, and in vivo. J Biol Chem.

[40] McLean PJ, Kawamata H, Hyman BT (2001). Alpha-synuclein-enhanced green fluorescent protein fusion proteins form proteasome sensitive inclusions in primary neurons. Neuroscience.

[41] Moree B, Yin G, Lazaro DF, Munari F, Strohaker T, Giller K, Becker S, Outeiro TF, Zweckstetter M, Salafsky J (2015). Small molecules detected by second-harmonic generation modulate the conformation of monomeric alpha-synuclein and reduce its aggregation in cells. J Biol Chem.

[42] Murphy DD, Rueter SM, Trojanowski JQ, Lee VM (2000). Synucleins are developmentally expressed, and alpha-synuclein regulates the size of the presynaptic vesicular pool in primary hippocampal neurons. J Neurosci.

[43] Olanow CW, Perl DP, DeMartino GN, McNaught KS (2004). Lewy-body formation is an aggresome-related process: a hypothesis. Lancet Neurol.

[44] Outeiro TF, Lindquist S (2003). Yeast cells provide insight into alpha-synuclein biology and pathobiology. Science.

[45] Outeiro TF, Putcha P, Tetzlaff JE, Spoelgen R, Koker M, Carvalho F, Hyman BT, McLean PJ (2008). Formation of toxic oligomeric alpha-synuclein species in living cells. PLoS One.

[46] Pasanen P, Myllykangas L, Siitonen M, Raunio A, Kaakkola S, Lyytinen J, Tienari PJ, Poyhonen M, Paetau A (2014). Novel alpha-synuclein mutation A53E associated with atypical multiple system atrophy and Parkinson’s disease-type pathology. Neurobiol Aging.

[47] Polymeropoulos MH, Lavedan C, Leroy E, Ide SE, Dehejia A, Dutra A, Pike B, Root H, Rubenstein J, Boyer R (1997). Mutation in the alpha-synuclein gene identified in families with Parkinson’s disease. Science.

[48] Proukakis C, Dudzik CG, Brier T, MacKay DS, Cooper JM, Millhauser GL, Houlden H, Schapira AH (2013). A novel alpha-synuclein missense mutation in Parkinson disease. Neurology.

[49] Rey NL, Steiner JA, Maroof N, Luk KC, Madaj Z, Trojanowski JQ, Lee VM, Brundin P (2016). Widespread transneuronal propagation of alpha-synucleinopathy triggered in olfactory bulb mimics prodromal Parkinson’s disease. J Exp Med.

[50] Rutherford NJ, Giasson BI (2015). The A53E alpha-synuclein pathological mutation demonstrates reduced aggregation propensity in vitro and in cell culture. Neurosci Lett.

[51] Sabate R, Rodriguez-Santiago L, Sodupe M, Saupe SJ, Ventura S (2013). Thioflavin-T excimer formation upon interaction with amyloid fibers. Chem Commun.

[52] Saito Y, Kawashima A, Ruberu NN, Fujiwara H, Koyama S, Sawabe M, Arai T, Nagura H, Yamanouchi H, Hasegawa M (2003). Accumulation of phosphorylated alpha-synuclein in aging human brain. J Neuropathol Exp Neurol.

[53] Singleton AB, Farrer M, Johnson J, Singleton A, Hague S, Kachergus J, Hulihan M, Peuralinna T, Dutra A, Nussbaum R (2003). alpha-Synuclein locus triplication causes Parkinson’s disease. Science.

[54] Spillantini MG, Schmidt ML, Lee VM, Trojanowski JQ, Jakes R, Goedert M (1997). Alpha-synuclein in Lewy bodies. Nature.

[55] Spillantini MG, Crowther RA, Jakes R, Hasegawa M, Goedert M (1998). alpha-Synuclein in filamentous inclusions of Lewy bodies from Parkinson’s disease and dementia with lewy bodies. Proc Natl Acad Sci U S A.

[56] Tenreiro S, Reimao-Pinto MM, Antas P, Rino J, Wawrzycka D, Macedo D, Rosado-Ramos R, Amen T, Waiss M, Magalhaes F (2014). Phosphorylation modulates clearance of alpha-synuclein inclusions in a yeast model of Parkinson’s disease. PLoS Genet.

[57] Tenreiro S, Rosado-Ramos R, Gerhardt E, Favretto F, Magalhaes F, Popova B, Becker S, Zweckstetter M, Braus GH, Outeiro TF (2016). Yeast reveals similar molecular mechanisms underlying alpha- and beta-synuclein toxicity. Hum Mol Genet.

[58] Tsigelny IF, Sharikov Y, Wrasidlo W, Gonzalez T, Desplats PA, Crews L, Spencer B, Masliah E (2012). Role of alpha-synuclein penetration into the membrane in the mechanisms of oligomer pore formation. FEBS J.

[59] Volles MJ, Lansbury PT (2007). Relationships between the sequence of alpha-synuclein and its membrane affinity, fibrillization propensity, and yeast toxicity. J Mol Biol.

[60] Wales P, Pinho R, Lazaro DF, Outeiro TF (2013). Limelight on alpha-synuclein: pathological and mechanistic implications in neurodegeneration. J Park Dis.

[61] Webb JL, Ravikumar B, Atkins J, Skepper JN, Rubinsztein DC (2003). Alpha-Synuclein is degraded by both autophagy and the proteasome. J Biol Chem.

[62] Weisberg SJ, Lyakhovetsky R, Werdiger AC, Gitler AD, Soen Y, Kaganovich D (2012). Compartmentalization of superoxide dismutase 1 (SOD1G93A) aggregates determines their toxicity. Proc Natl Acad Sci U S A.

[63] Winner B, Jappelli R, Maji SK, Desplats PA, Boyer L, Aigner S, Hetzer C, Loher T, Vilar M, Campioni S (2011). In vivo demonstration that alpha-synuclein oligomers are toxic. Proc Natl Acad Sci U S A.

[64] Xu W, Tan L, Yu JT (2015). Link between the SNCA gene and parkinsonism. Neurobiol Aging.

[65] Zarranz JJ, Alegre J, Gomez-Esteban JC, Lezcano E, Ros R, Ampuero I, Vidal L, Hoenicka J, Rodriguez O, Atares B (2004). The new mutation, E46K, of alpha-synuclein causes Parkinson and Lewy body dementia. Ann Neurol.

