# Supplementary figures and images for: The effects of the novel A53E alpha-synuclein mutation on its oligomerization and aggregation
Source: Acta Neuropathol Commun. 2016 Dec 9;4:128. doi: 10.1186/s40478-016-0402-8 (PMC5148884; doi:10.1186/s40478-016-0402-8)

**S1.1**

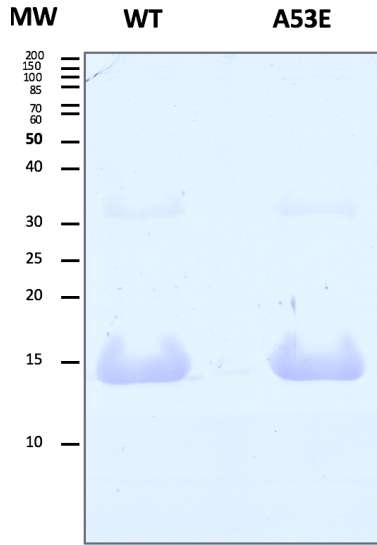

**S1.2**

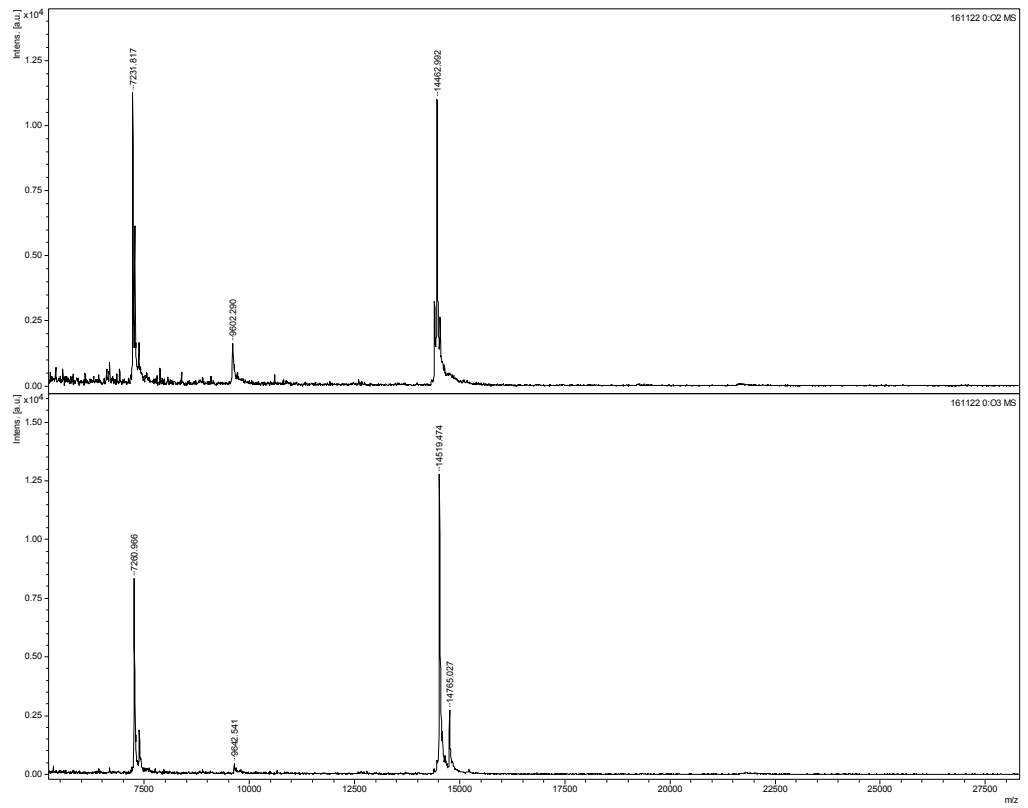

**S2.1**

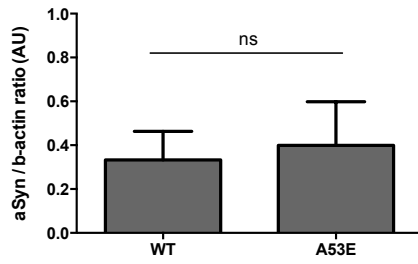

**S2.2**

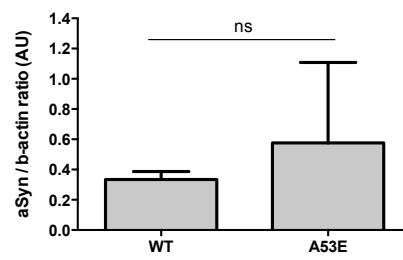

**S3.1**

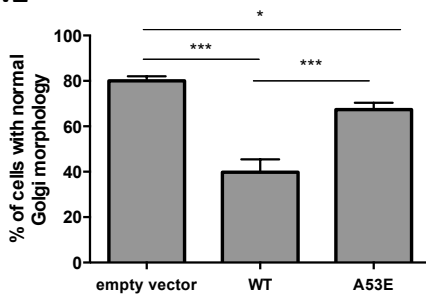

**S3.2**

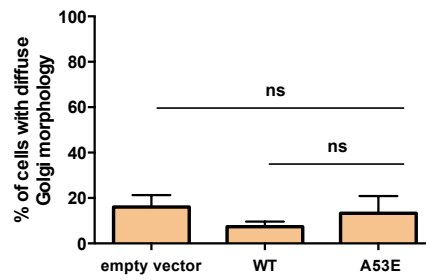

**S3.3**

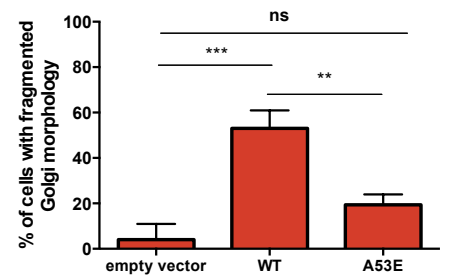

**S3.4**

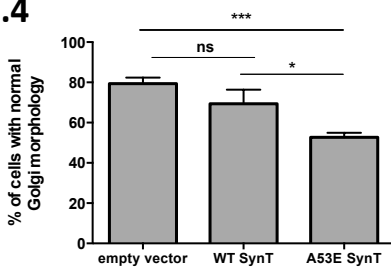

**S3.5**

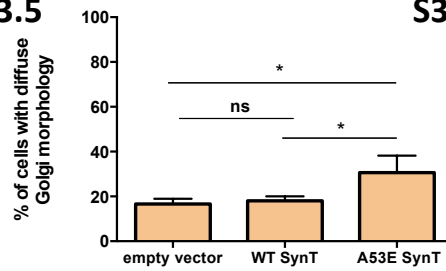

**S3.6**

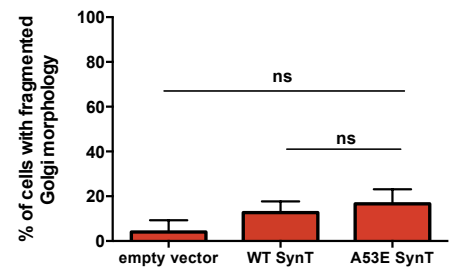

Supplement: Additional file 1: Figure S1.1. — Biochemical characterization of WT and A53E recombinant aSyn. Analysis of purified WT and A53E aSyn variants by SDS-PAGE (18%) stained with Coomassie Brilliant Blue. S1.2. MALDI-TOF mass spectrometry analysis of purified WT (up) and A53E (down) aSyn variants. A MW of 14462.98 Da was obtained for WT (theoretical of 14460.16 da) and of 14519.47 Da for A53E (theoretical of 14518.19 Da) aSyn variants. The peaks of 7231.82 and 7260.97 Da correspond to the M+2 ions of WT and A53E aSyn, respectively. Figure S2.1 and S2.2. Immunoblot quantifications. Levels of VN-aSyn (S2.1) and aSyn-VC (S2.1). n=3. Figure S3.1-S3.3. Morphological analysis of Golgi apparatus in the aSyn BiFC system. The morphology of the Golgi was analyzed as normal (S3.1), diffuse (S3.2) and fragmented (S3.3). One-way ANOVA with post-hoc Tukey’s test (*p<0.05, **p<0.01, ***p<0.001). n=3. Figure S3.4-3.6 Morphological analysis of Golgi apparatus in the aSyn aggregation model. Transfected cells were analyzed according to the morphology of the Golgi: normal (S3.4), diffuse (S3.5) and fragmented (S3.6) One-way ANOVA with post-hoc Tukey’s test (*p<0.05, **p<0.01, ***p<0.001). n=3. (PDF 389 kb) [file 40478_2016_402_MOESM1_ESM.pdf]
